# Contribution of histone variants to aneuploidy: a cancer perspective

**DOI:** 10.3389/fgene.2023.1290903

**Published:** 2023-11-23

**Authors:** Denise Ragusa, Paola Vagnarelli

**Affiliations:** College of Health, Medicine and Life Sciences, Department of Life Sciences, Brunel University London, London, United Kingdom

**Keywords:** histone, variant, aneuploidy, chromosome, cancer, database

## Abstract

Histone variants, which generally differ in few amino acid residues, can replace core histones (H1, H2A, H2B, and H3) to confer specific structural and functional features to regulate cellular functions. In addition to their role in DNA packaging, histones modulate key processes such as gene expression regulation and chromosome segregation, which are frequently dysregulated in cancer cells. During the years, histones variants have gained significant attention as gatekeepers of chromosome stability, raising interest in understanding how structural and functional alterations can contribute to tumourigenesis. Beside the well-established role of the histone H3 variant CENP-A in centromere specification and maintenance, a growing body of literature has described mutations, aberrant expression patterns and post-translational modifications of a variety of histone variants in several cancers, also coining the term “oncohistones.” At the molecular level, mechanistic studies have been dissecting the biological mechanisms behind histones and missegregation events, with the potential to uncover novel clinically-relevant targets. In this review, we focus on the current understanding and highlight knowledge gaps of the contribution of histone variants to aneuploidy, and we have compiled a database (HistoPloidyDB) of histone gene alterations linked to aneuploidy in cancers of the The Cancer Genome Atlas project.

## 1 Structural and functional features of histones and histone variants

**FIGURE 1:**
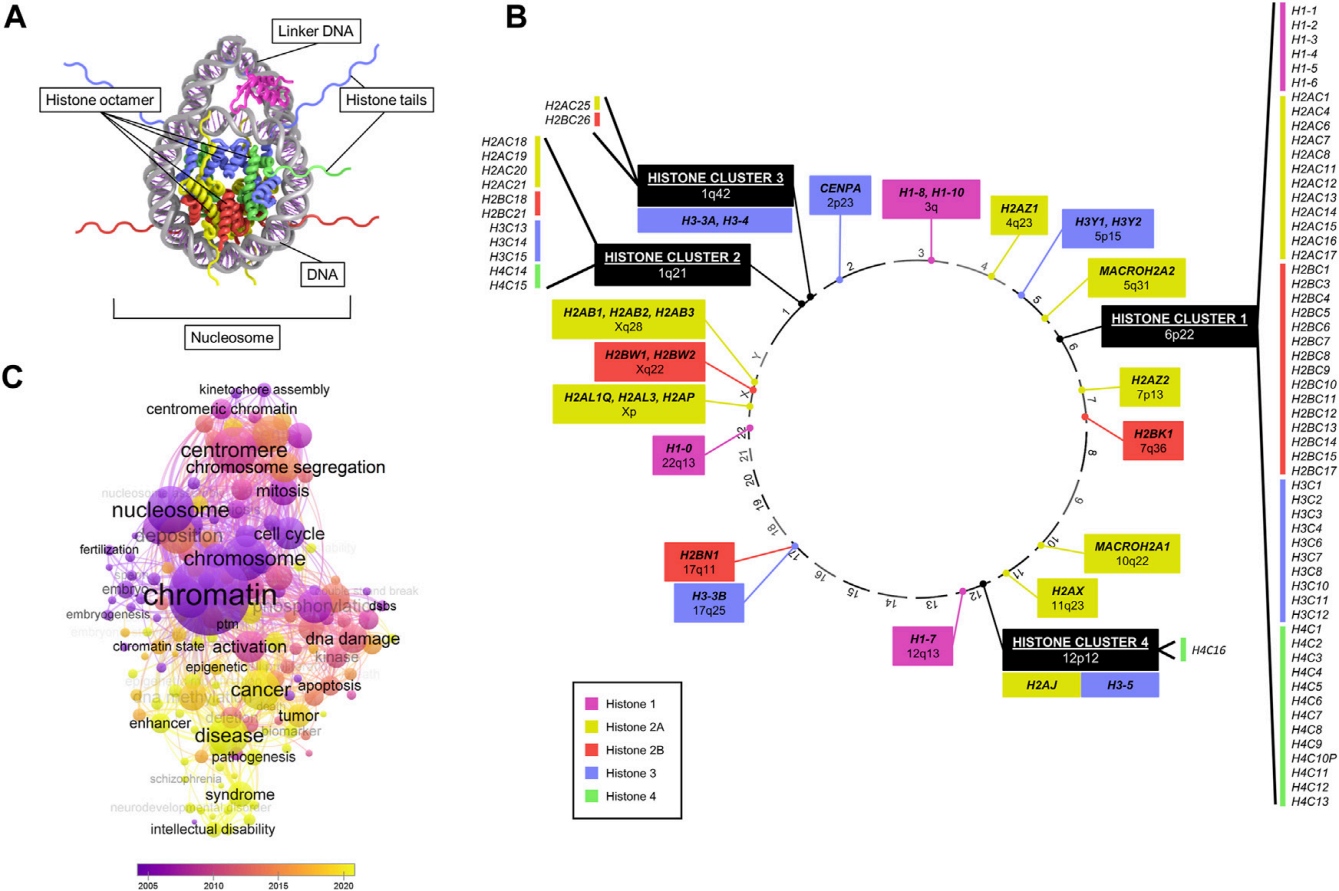
Overview of canonical and variant histone genes. **(A)** Crystal structure of a nucleosome, consisting of histone proteins (forming an octamer) wrapped by DNA. Data adapted from HistoneDB 2.0 (Draizen et al., 2016). **(B)** Chromosomal location (numbered outside of circle) of all human histone genes. Colours represent different core histones *H1, H2A, H2B, H3*, and *H4*. Histone variants are marked in bold text. **(C)** Bubble plot of numbers of enriched keywords co-occurring in publications searched by “*histone variant*” in PubMed by year. Generated with VOSviewer.

Histones are fundamental protein components of the nucleosome, which constitutes the basic unit of chromatin in eukaryotes. Nucleosomes are built as octamers of core histones, namely, H2A, H2B, H3, and H4, and are wrapped by approximately 146 bp of DNA (Figure 1A). Histone H1, known as linker histone, is responsible for modulation of chromatin structure between nucleosomes (Luger et al., 1997). Core histones H2A, H2B, and H3, as well as linker histone H1, have paralogues that often differ in few amino acid residues, known as histone variants. Recently, a larger repertoire of histone genes and variants has been uncovered, including an *H4* variant *H4*.*G* and several pseudogenes with putative regulatory functions (Long et al., 2019; Seal et al., 2022). As pictured by Maze et al. (Maze et al., 2014), “every amino acid matters” for histone variants, as minor changes in amino acid composition can produce profound changes in functional properties. Histone variants are incorporated into the nucleosome to confer specific structural features to accomplish biological outcomes. In fact, histones and their variants regulate a plethora of functions, including regulation of DNA replication and repair, chromatin organisation, transcription, and chromosome segregation (Bernstein and Hake, 2006; Martire and Banaszynski, 2020). The mechanisms behind the “decision” of which histones to incorporate within the correct spatio-temporal window is still an active research question, which is dependent on a tight regulation of histone availability and the coordination with chaperones and histone modifying enzymes, promoting or disrupting the deposition of certain variants over others (Mendiratta et al., 2019).

In humans, histone genes are transcribed from distinct genomic loci (Draizen et al., 2016; Seal et al., 2022) (Figure 1B). The majority of canonical histones are found at a specific locus at 6p22 known as “histone cluster 1”. Three other clusters are located at 1q21, 1q42, and 12p12, harbouring the remainder of canonical histones and a few variants (*H3.3A*, *H3.4*, *H3*.*5*, and *H2A.J*). All other histone variants map at various cytobands (Figure 1B). A key difference between canonical and variant histones is the difference in expression during the cell cycle, as canonical histones are replication-dependent, while most of the variants are not (Mei et al., 2017). In addition, almost all canonical histone transcripts do not contain introns or poly(A) tails unlike variants (Maze et al., 2014).

Structurally, core histone proteins consist of a conserved histone-fold domain and a protruding N-terminal tail domain that is the main, yet not exclusive, site of post-translational modifications (PTM). In addition to the variability in nucleosome composition conferred by the incorporation of canonical and variant histones, PTMs also add a further layer of diversity to physical properties and thus functional variability (Bernstein and Hake, 2006; Cavalieri, 2021). Histone variants can possess unique structural features compared to their canonical counterpart, although the majority of variants only present single amino acid substitutions (Maze et al., 2014; Martire and Banaszynski, 2020). The families of H1 and H2A variants show the highest diversity in structural composition compared to H2B and H3 (Maze et al., 2014; El Kennani et al., 2018; Prendergast and Reinberg, 2021).

Due to their role in regulating key cellular processes, the potential of histone variants in contributing to tumourigenic processes has been of great interest (Buschbeck and Hake, 2017). Analysis of PubMed publications featuring “*histone variants*” revealed an association with a wide range of cellular functions (e.g., cell cycle, mitosis, chromosome segregation, aneuploidy, DNA damage), and a growing association with diseases, notably cancer, in recent years (Figure 1C). In particular, research interest has accrued since the discovery of specific *H3* histone driver mutations in glioma disrupting a key methylation site, which coined the term “oncohistone” (Schwartzentruber et al., 2012; Sturm et al., 2012; Wu et al., 2012; Pathania et al., 2017; Nacev et al., 2019). Since then, a large body of literature has grown on histone mutations implicated in several malignancies, including lymphoma (Lohr et al., 2012), chrondroblastoma (Behjati et al., 2013), ovarian cancer (Zhao et al., 2016), and head and neck tumours (Papillon-Cavanagh et al., 2017).

The contribution of histone deregulation to carcinogenesis is multi-faceted and can include effects on gene expression regulation, cell identity, and genomic stability via chromosome integrity and DNA damage response (Buschbeck and Hake, 2017; Ferrand et al., 2020). Genomic instability is tightly correlated, and often precedes, aneuploidy–a hallmark of cancer, broadly defined as numerical and structural aberrations of chromosomes (Sheltzer, 2013; Ben-David and Amon, 2020). Several mechanisms can produce aneuploidy, depending on the type, including defective mitotic machinery, unresolved DNA damage, and centromeric and telomeric dysfunctions (Orr et al., 2015); however, the exact aetiology and contribution of aneuploidy to cancer initiation and progression remains unclear (Molina et al., 2021). The centromeric histone H3 variant CENP-A has been extensively studied as a gatekeeper of centromere maintenance (Renaud-Pageot et al., 2022), however a wider range of variants of other histone families are gaining similar interest and appear to contribute to chromosome stability (Ferrand et al., 2020; Prendergast and Reinberg, 2021; Sales‐Gil et al., 2021; Oberdoerffer and Miller, 2023). In particular, histone variants CENP-A, H3.3, H2A.Z, and macroH2A play a crucial role in defining functional chromatin regions (i.e., centromeres, pericentromeric regions, telomeres, inactive X chromosomes), which affect the ability of the cell to faithfully segregate chromosomes by appropriately assembling kinetochore machinery or maintaining telomere length (Ferrand et al., 2020).

In this review, we focus on the contribution and interplay between core histones and histone variants to aneuploidy, by a comprehensive overview of histone variants alterations in cancer. By analysing publicly available cancer data from the Cancer Genome Atlas (TCGA) project, we compiled HistoPloidyDB–a database of correlations between histone gene alterations and aneuploidy (available at https://vagnarelli-lab.github.io/HistoPloidyDB/).

## 2 Dysregulated expression of histone genes affecting chromosomal stability

**FIGURE 2:**
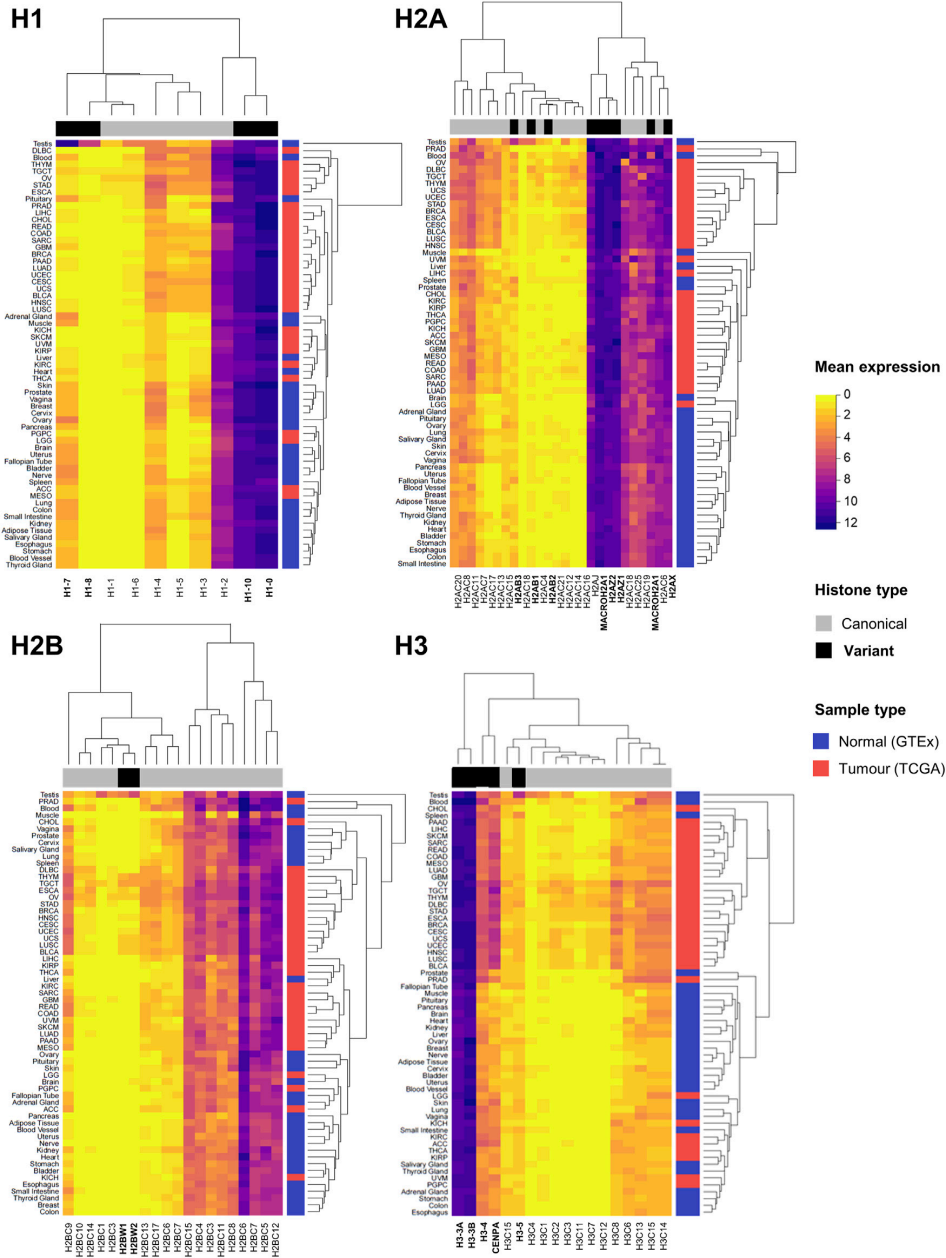
Expression overview of histone genes in normal (GTEx) and tumour (TCGA) sites. Heatmaps showing the mean mRNA expression of histone genes in units of log2(norm_count+1) by histone families H1, H2A, H2B, and H3. Hierarchical clustering of Euclidean distances defines similarities and differences in expression patterns, both by canonical and variant genes (indicated on the horizontal side bar in grey and black, respectively), and by site (normal tissue in blue and tumour tissue in red on vertical side bar). Normalised expression data by TOIL pipeline was obtained from University of California Santa Cruz (UCSC) Xena public repository (Goldman et al., 2020).

Bioinformatics analyses have identified histone gene deregulation as a common core regulatory component at pan-cancer level, together with chromatin remodeling, cell cycle, and immune response (Knaack et al., 2014). Figure 2 illustrates the expression levels of canonical and variant histones in tumour samples of TCGA and healthy tissues from the Genotype-Tissue Expression (GTEx) database, highlighting distinct patterns of expression between canonical and variant genes, as well as by site. Generally, the patterns of expression are distinct in tumours compared to healthy samples, with tissue-specific variability, for both canonical and variant histones. It is known that the regulation of canonical histone gene expression from the major histone clusters is complex, and that the expression of few canonical genes accounts for the majority of transcripts for each core histone (Mei et al., 2017; Flaus et al., 2021). Germline-specific variant histones display unique expression profiles (Doenecke et al., 1997), which is particularly evident in non-cancerous testes samples (Figure 2).

**FIGURE 3:**
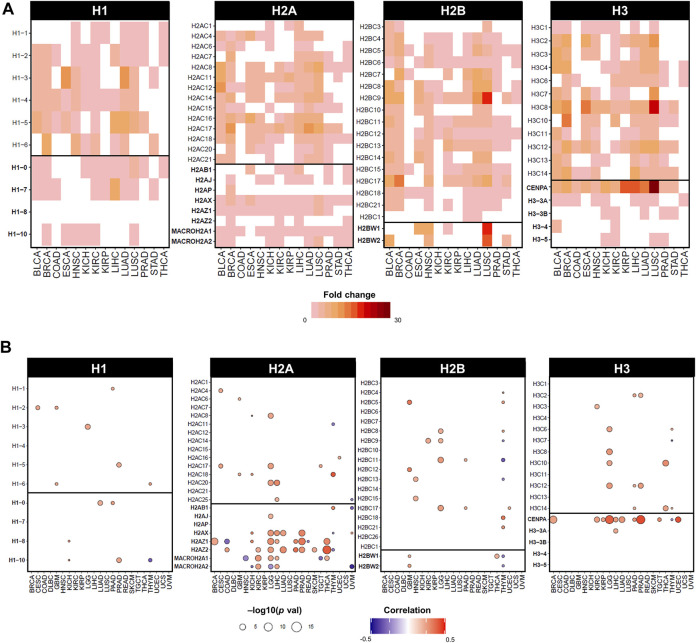
Dysregulated expression of histone genes in cancers of TCGA cohort. **(A)** Statistically significant fold changes in mRNA expression between tumour tissue and matched normal tissue from TCGA. Fold change values were calculated on normalised and batch corrected RSEM values in tumour sites with more than 10 matched tumor and normal samples. The fold change was computed by GSCA (Liu et al., 2023) and calculated as mean(tumour)/mean(normal), statistical significance determined by *p*-value (<0.05) by *t*-test. **(B)** Statistically significant correlations by Pearson correlation coefficients and statistical significance by *p*-value computed on mRNA expression in RSEM and Aneuploidy Score (i.e., number of altered chromosome arms), obtained from cBioPortal on TCGA cancers from PanCancerAtlas (Cerami et al., 2012; Gao et al., 2013).

In cancer, histone genes have a two-fold likelihood of overexpression compared to other genes, however their underexpression is uncommon (Shah et al., 2014; Ferrand et al., 2020; Ghiraldini et al., 2021). Expression data from the TCGA database shows a widespread overexpression of both canonical and variant genes between tumour tissue and matched surrounding healthy tissues, as depicted in Figure 3A by statistically significant fold changes in expression. The upregulation of replication-dependent histones may simply indicate a requirement for higher amounts of histones in proliferating cells undergoing high rates of replication, like cancer cells; however, an imbalanced transcriptional activity of histones can also cause or contribute to oncogenic programmes, as several lines of evidence have highlighted how overexpression of histones can adversely affect gene expression regulation and genomic stability that fuels cancer cells (Miles et al., 2019; Bruhn et al., 2022).

In fact, the expression of specific histone genes correlates with aneuploidy levels in several cancers of the TCGA cohort, notably *H2A* and *H3* variants, but also several canonical histones (Figure 3B). Although observational, dysregulation of histone genes occurs in cancers with evidence of genomic/chromosomal instability. For instance, canonical linker histone *H1*.*1* is downregulated and differentially methylated in childhood acute lymphoblastic leukaemia (ALL) with hyperdiploid karyotypes (Davidsson et al., 2009). The canonical histones *H1*.*2*, *H2AC18*, *H2BC6*, and *H2BC21* are upregulated in multiple myeloma, which has an aneuploidy rate of 90% of cases (Zhan et al., 2002). Cancer cells with higher levels of chromosomal instability showed lower levels of expression of canonical *H3* and histone variants *H1*.*10*, *H2A.X*, and *H2A.Z* (Roschke and Kirsch, 2010). Moreover, the entire 6q22 histone locus is overexpressed in aggressive subtypes of liposarcoma that displays high rates of copy number abnormalities (Yoo et al., 2021).

In support of these observations, a large body of literature has described phenotypes of mitotic defects linked to alterations in histone levels. Loss of histone variant H3.3 in murine fibroblasts resulted in defects in chromosome alignment in mitosis and production of multi-lobed nuclei and micronuclei, as well as decreased proliferation and increase in cell death (Ors et al., 2017). Other studies also demonstrated that loss of H3.3 in mouse embryonic stem cells and mouse embryonic fibroblasts produces severe mitotic defects in the form of lagging chromosomes and anaphase bridges (Jang et al., 2015), and that H3.3 is necessary to ensure mitotic fidelity in early mouse development to prevent aneuploidy (Bush et al., 2013; Lin et al., 2013). In p53 deficient backgrounds, these chromosomal defects are accentuated, with higher rates of numerical and structural aneuploidies (Jang et al., 2015). H3.3 was also linked to chromosome instability by numerical aneuploidy by overexpression of Aurora B, which causes an increase in mitotic phosphorylation of H3.3 (Ota et al., 2002). Loss of H1.1 in *Xenopus* was shown to cause anaphase segregation defects, due to abnormally elongated chromosomes (Maresca et al., 2005). Overall, these studies lay the ground for exploring the dysregulation of these variants and their contribution to genomic instability of tumours.

### 2.1 Histone excess compromises the balance of histone availability and incorporation

A number of mechanisms have been proposed to account for the excessive supply of histones that can compromise chromosomal stability (Singh et al., 2010; Miles et al., 2019). An accumulation of histones, regardless of canonical or variant forms, can disrupt the balance between nucleosome-bound and free histones, and outcompete the incorporation of the required histones. In *S. cerevisae*, excessive canonical histone levels result in increased rates of chromosome loss (Meeks-Wagner and Hartwell, 1986; Gunjan and Verreault, 2003; Singh et al., 2010). Also in yeast, prolonged exposure to high levels of H2A and H2B histones produces mitotic defects that can results in whole genome duplication (WGD) via endomitosis, concomitant with a reduced incorporation of the H2A.Z variant into chromatin due to an outcompetition mechanism (Miles et al., 2018). Similarly, excessive levels of canonical H3 leads to chromosome loss by interfering with the normal deposition of the centromere-specifying variant CENP-A (Castillo et al., 2007; Au et al., 2008).

In addition, owing to their basic composition, histones are prone to engage in unwanted interactions with acidic cellular components and negatively charged macromolecules (Singh et al., 2009; Mendiratta et al., 2019). Therefore, an excessive reservoir of histones can induce deleterious effects by inappropriate interactions with histone chaperones, modifying enzymes, or nucleic acids (Gunjan and Verreault, 2003; Singh et al., 2010). In fact, in yeast cells, overexpression of histone chaperones can counteract the cytotoxic effects of histone excess, which include slower proliferation, DNA damage sensitivity, and genomic instability via chromosome loss (Gunjan and Verreault, 2003). Histone chaperones are tightly linked to histone function, as their role in deposition and histone level control ensures an appropriate balance of incorporation and exchange (Hammond et al., 2017), which can be compromised during carcinogenesis. Like histone genes, histone chaperone genes are also dysregulated in cancer, including the H3-H4-cargoing chaperones *ASF1*, *HIRA*, *CAF-1*, *DAXX*, and *DEK*, the H3-H4 and H2A-H2B chaperone *FACT*, and the CENP-A-H4 chaperone *HJURP* (Ray-Gallet and Almouzni, 2022; Zhao et al., 2022). In terms of contribution to aneuploidy, several studies have shown the importance of histone chaperones in maintaining chromosomal stability, including increased levels of aneuploidy upon loss of *FACT* (Prendergast et al., 2020), *ASF1* (Horard et al., 2018) and *HJURP* (Filipescu et al., 2017), mitotic defects and micronuclei following overexpression of *DEK* (Matrka et al., 2015), and expansion of ribosomal DNA (rDNA) repeats in absence of ASF1 (Houseley and Tollervey, 2011), which can be a source of chromosomal aberrations if inappropriately resolved (Daniloski et al., 2019). Clinically, the expression of *CAF-1* was found to be correlated with ploidy status in breast cancer, as well as other clinical features of disease progression (Polo et al., 2004). In cancer cell lines, the induction of metastatic programmes resulted in an increased occupancy of the H3.3 variant, with a concomitant reduction of other canonical histones. However, this effect was dependent on *CAF-1*, as demonstrated by the induction of the metastatic phenotype upon depletion of the chaperone, but not by *H3.3* overexpression alone (Gomes et al., 2019). This synergism between chaperones and histones is also supported by a co-operative effect of overexpression of *H3.3A* and *ASF1* inducing proliferation of pancreatic beta cells (Paul et al., 2016). Therefore, the deleterious chromosomal and oncogenic effects resulting from a dysregulated availability of histones are also interlinked with their chaperones.

### 2.2 Environment-driven changes in histone expression levels

Exposure to carcinogens, including benzene (McHale et al., 2009) and Human T-Lymphotropic Virus Type-1 (HTLV-1) (Bogenberger and Laybourn, 2008), as well as treatments with DNA damage-inducing agents such as ionising radiation, Adriamycin, and cisplatin (Su et al., 2004), have also been linked to changes in canonical histone levels and induction of genomic instability.

One mechanism that has been proposed for the link between environmental exposure and oncogenic histone activity involves aberrant polyadenylation of histone *H3*.*1* transcripts (Brocato et al., 2015; Chen et al., 2020). Replication-dependent histones are not polyadenylated at the 3′ to ensure degradation that is required to maintain normal replication-dependency of canonical histones. Instead, canonical histones possess a stem-loop structure at the 3′ responsible for post-translational processing. When exposed to arsenic, *H3*.*1* mRNA acquires a poly(A) tail, which increases its stability and consequent protein abundance (Brocato et al., 2015; Chen et al., 2020). The excessive availability of H3.1 outcompetes the H3.3 variant from chromatin regulatory regions, resulting in changes in gene expression, cell cycle dysfunctions, and aneuploidy (Chen et al., 2020). The authors highlighted the displacement of H3.3 as a core component of transformation by arsenic, also supported by genetic manipulation of the *H3.3* variant alone; in fact, transformation was achieved by knock-down of *H3.3* and overexpression of *H3.3* could rescue arsenic-dependent transformation (Chen et al., 2020). Exposure to nickel also increases the polyadenylation of *H3*.*1* transcripts, creating a “histone excess” problem that leads to inappropriate incorporation of free histones onto chromatin, inducing genomic instability and transformation (Jordan et al., 2017). The aberrant polyadenylation of canonical histones can also results from decreased levels of the stem-loop binding protein (SLBP), which normally binds to the 3′ stem-loop structure to correctly coordinate histone degradation (Brocato et al., 2015; Jordan et al., 2017). In *Drosophila*, dysfunctional SLBP leads to chromosomal instability, including chromosomal breaks and numerical aneuploidy, via the accumulation of histones with poly(A) tails (Salzler et al., 2009).

The observed reduction in histone levels (both at transcriptional and protein levels) has also been linked to the response to the DNA damage caused by the carcinogenic agents, which triggers DNA damage response mechanisms and a block of DNA synthesis leading to genomic instability (Su et al., 2004). While this may contradict the effects of excess histone genes, these mechanisms have been attributed to DNA hypomethylation, in a similar fashion to loss of imprinting, which are also causative of deleterious genomic effects, including aneuploidy (Eden et al., 2003; Bogenberger and Laybourn, 2008). In the study on HTLV-1, no effects were detected in ploidy in the infected cells (Bogenberger and Laybourn, 2008), however the appearance of chromosomal anomalies may be a subsequent event in response to an increasingly instable genome. For example, mutations of *H3* variants in giant cell tumour of the bone were shown to predate the development of karyotype defects (Fittall et al., 2020). Downregulation of canonical histones (*H2AC8*, *H2AC16*, *H2BC9*, *H2BC13*, *H3C6*, *H3C7*, and *H4C6*) has been reported to increase genomic instability in giant cell tumour of bone stromal cells (Lau et al., 2017). Therefore, the role of histone deregulation is likely to be dynamic and follow multiple mechanisms.

Another example of environmental influence on histone activity is the modulation of karyotype by *C. albicans* as a resistance mechanism to antifungal drugs. The induction of numerical and structural aneuploidy is a common mechanism of stress response in the pathogen (Selmecki et al., 2009). *C. albicans* possesses two *H2A* homologues, of which one (*H2A.1*) has evolutionarily lost a key Bub1 phosphorylation site for maintenance of faithful chromosome segregation. Indeed, *H2A.1* promotes the occurrence of aneuploidy through antimycotic treatment, which also results in depletion of CENP-A at centromeres (Brimacombe et al., 2018). This is also in agreement with increased chromosomal instability in yeast H2A mutants at C-terminal sites of Bub1 phosphorylation (Kawashima et al., 2010). Karyotype evolution is an important phenomenon in the context of cancer, with consequences on disease progression and resistance to treatment. In particular, the process of polyploidisation can be induced during differentiation or in response to stressors, including chemotherapeutic drugs, ionizing radiation, hypoxia, or reactive oxygen species (Was et al., 2022). Upregulation of both canonical (*H1*, *H2A*, *H2B*, and *H3*) and variant histones (*H1*.*0*, *H2A.X* and *H3.3B*) was identified in a mouse erythroleukaemia cell line with a tetraploid phenotype acquired through resistance to differentiation (Fernández-Calleja et al., 2017). Changes in histone composition have also been reported in murine trophoblast giant cells (TGC), which are placental cells with large polyploid nuclei generated by endoreplication. Compared to the undifferentiated (non-polyploid) counterparts, TGCs exhibited a reduction in expression of canonical histones but maintenance of high levels of histone variants including *H2A.X*, *H2A.Z*, and *H3.3* (Hayakawa et al., 2018). Similarly, several canonical histones (*H2AC8*, *H2AC16*, *H2BC9*, *H2BC13*, *H3C7*, *H4C6*) were found to be upregulated in polyploid giant cancer cells derived from MCF7 breast cancer lines (Buehler et al., 2022), and rat hepatocytes treated with the polyploidy-inducing carcinogen thioacetamide showed a hypermethylation and downregulation of canonical *H2AC1* (Mizukami et al., 2017). Similarly, the expression of histone variant *H1*.*0* increases from untransformed to highly transformed astrocytes, with a direct correlation with aneuploidy status (Caradonna et al., 2020), further suggesting a link between environmental stresses and histone deregulation in the context of transformation.

### 2.3 Overexpression of histone variants causing centromeric dysfunction

The centromeric *H3* variant *CENP-A* is overexpressed in a plethora of human malignancies [recently reviewed by (Renaud-Pageot et al., 2022)], while its under-expression has not been reported. Physiologically, CENP-A participates in centromere maintenance by structurally and functionally defining centromeric regions (Stirpe and Heun, 2023). In virtue of their pivotal role as docking stations for the kinetochore, centromeres and chromosome segregation are tightly linked, therefore an association between CENP-A with aneuploidy is not surprising. It has been demonstrated that overexpression of *CENP-A* is responsible for its aberrant centromeric localisation, chromosomal instability and aneuploidy (Giunta et al., 2021; Shrestha et al., 2021). *CENP-A* was found to be overexpressed in colorectal cancer patient samples showing a mislocalisation to non-centromeric regions (Tomonaga et al., 2003), indicating that dysregulation at the transcriptional level can lead to an aberrant centromere specification and subsequently erroneous assembly of mitotic machinery and missegregation.

Functional centromeres also rely on histone variants other than CENP-A, principally the H2A.Z variant. Both CENP-A and H2A.Z occupy specific regions of centromeres and contribute to their structure and function (Greaves et al., 2007; Boyarchuk et al., 2014). The association of H2A.Z with mitotic fidelity, and particularly with the co-ordination of kinetochore attachment, has been well documented (Krogan et al., 2004; Rangasamy et al., 2004; Greaves et al., 2007; Hou et al., 2010; Kawashima et al., 2010; Sharma et al., 2013; Boyarchuk et al., 2014; Sales‐Gil et al., 2021). In *Schizosaccharomyces pombe*, chromosome stability is ensured via co-ordination of CENP-A deposition and requires the presence of H2A.Z-homologue *pht1* (Ahmed et al., 2007). Earlier studies in *S. pombe* reported that deletions in *pht1* resulted in reduced chromosome segregation fidelity and chromosome loss (Carr et al., 1994), which was also observed in later studies where depletion of *H2A.Z* caused genomic instability and chromosome segregation defects including lagging chromosomes, chromatin bridges (Rangasamy et al., 2004), and chromosome losses (Krogan et al., 2004; Hou et al., 2010). Mislocalisation or overexpression of *H2A.Z* has also been shown to cause defective centromeres and increased aneuploidy in yeast with defective INO80 chromatin remodeling complex (Chambers et al., 2012). Similarly, deletion of *H2A.Z* resulted in chromosome losses and aneuploidy, together with centromeric defects involving CENP-A (Ling and Yuen, 2019). In addition to mitotic defects, the *H2A.Z*-deleted fission yeast strains described by Carr et al. (Carr et al., 1994) also exhibited lower proliferative rates, abnormal colony morphology, and improved heat shock resistance, which agrees with H2A.Z’s functions beyond centromeric regulation, including transcriptional regulation that can produce more “global” phenotypic effects.

The H2A variant family comprises of several functionally distinct members involved in a variety of cellular processes (Oberdoerffer and Miller, 2023). Interestingly, expression of *H2A* variants shows the strongest correlations with aneuploidy second to *CENP-A* (Figure 3B), and *H2AZ.2* and *H2A.X* have been identified as top 70 genes in signatures defining functional aneuploidy and chromosomal instability at pan-cancer level (Carter et al., 2006). Although the H2A.Z variant has been investigated as a single entity, recent studies have uncovered that its two paralogues H2AZ.1 and H2AZ.2 accomplish distinct functions (Lamaa et al., 2020; Sales‐Gil et al., 2021). H2A.Z.1 is mainly involved in transcriptional and cell cycle regulation, whereas H2A.Z.2 is required for faithful chromosome segregation (Sales‐Gil et al., 2021). These differences are also reflected in the involvement of specific variants in the pathogenesis of a number of malignancies (Vardabasso et al., 2015; Tang et al., 2020; Han et al., 2022; Zheng et al., 2022). Gastroesophageal adenocarcinoma cells lines showed a positive correlation between high expression of *H2A.X* and features of chromosomal instability and WGD events (Sahgal et al., 2023). Clinically, *H2A.X* transcriptional levels had prognostic value in patient cohorts and could predict drug sensitivity to compounds targeting DNA damage response in cell lines (Sahgal et al., 2023). Furthermore, also belonging to the H2A variant family, macroH2A.1 and macroH2A.2 are two peculiar variants characterised by the presence of a macro domain that functions as a binding site for macromolecule docking. They both function in orchestrating gene expression regulation and genome integrity by managing DNA repair and replication stress (Kim et al., 2018). In mice, loss of the splice variant macroH2A.1.2 caused an increase in micronuclei and aneuploidy mainly of the X chromosome, but also on other autosomes albeit at a lower rate, caused by inefficient repair of under-replicated DNA (Sebastian et al., 2020).

### 2.4 Histone expression dysregulation in ageing-related aneuploidy

Some studies also indicate a possible role of histone alterations in ageing-related aneuploidy. Ageing, in the form of chronological age as well as senescence phenotypes, is associated with chromosomal abnormalities that can promote the development of malignancy (Tanaka et al., 2018). Reduction of canonical histone levels and accumulation of histone variants has been associated with ageing [reviewed by Yi and Kim (Yi and Kim, 2020)]. In particular, histone variants belonging to the H3 and H2A families have been reported at increased levels in aged or senescent cells, namely, *H3.3*, *H2A.Z*, *H2A.J*, and *macroH2A* (Rogakou and Sekeri–Pataryas, 1999; Kreiling et al., 2011; Maze et al., 2015; Contrepois et al., 2017; Tvardovskiy et al., 2017; Stefanelli et al., 2018). Histone variant *H3.3* has been identified as a candidate gene for ageing-related aneuploidy by differential gene expression between young and aged fibroblasts (Geigl et al., 2004). Moreover, high levels of histone variant *H2A.J* are associated with senescence and a promotion of inflammatory signatures (Contrepois et al., 2017; Isermann et al., 2020), which is a similar phenotype observed through depletion of *H2A.Z.1* leading to senescence (Sales‐Gil et al., 2021). In yeast, ageing produced a marked reduction in nucleosome occupancy, resulting in a widespread transcriptional activation, genomic instability, and an increased frequency of chromosomal translocations (Hu et al., 2014). Dysfunctional H1.4 histone activity is linked to a premature senescent phenotype, accompanied by high degree aneuploidy (Flex et al., 2019). Also belonging to the linker histone family, histone variant H1.8 functions in ensuring meiotic maturation in oocytes (Furuya et al., 2007) and its expression levels are downregulated in aged oocytes in comparison with young ones (Jiao et al., 2012). An increased expression of the canonical histone *H2AC1* has also been documented in older oocytes (Ntostis et al., 2022), while canonical histone *H2BC3* is upregulated in aneuploid *in vitro* fertilisation (IVF) embryos (Huang et al., 2021; Yaacobi-Artzi et al., 2022). Interestingly, aneuploid oocytes have been shown to express a lower level of the histone-like protein HILS1 (Fragouli et al., 2010). In the context of ageing, an inadequate supply of histones can result in altered epigenetic marks, with consequences on the maintenance of transcriptional and genomic control; in fact, ageing is associated with dysregulated epigenetic patterns (Wang et al., 2018), including aberrant acetylation patterns in ageing-related aneuploidy in oocytes specifically (Akiyama et al., 2006). In addition to a dysregulation in expression of *H2A* and *H3* variants, aged fibroblast also present differential expression of genes involved in histone modifications (Geigl et al., 2004).

## 3 Functional consequences impacting ploidy by mutations of histone genes—PTMs and beyond

**FIGURE 4:**
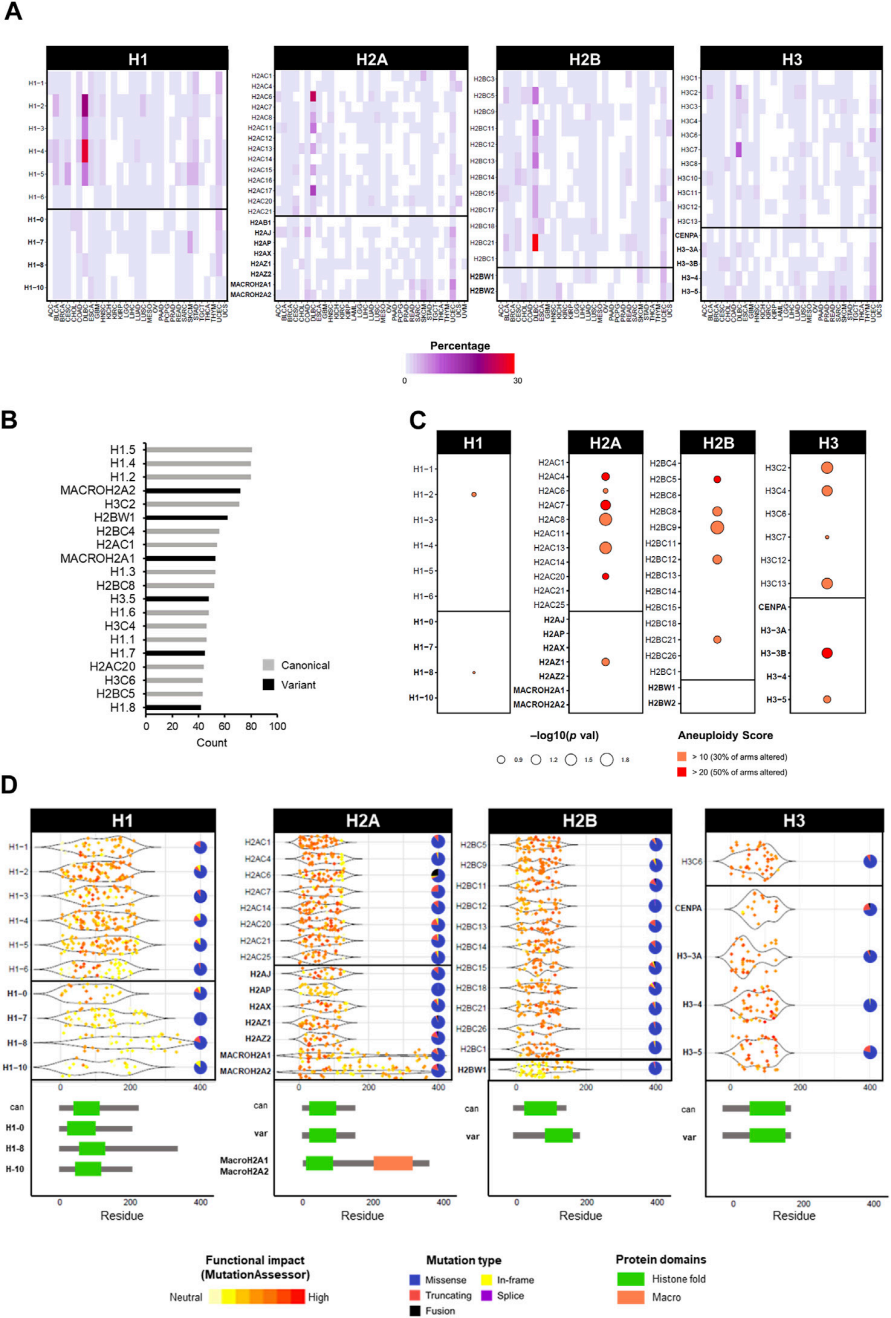
Overview of mutations in histone genes in cancers of the TCGA database. **(A)** Frequency of mutations as single nucleotide variations in TCGA cancers in percentage. Mutation data was obtained from cBioPortal in TCGA cancers from PanCancerAtlas (Cerami et al., 2012; Gao et al., 2013). **(B)** Top 20 mutated histone genes by number of cases across all cancers, distinguishable by canonical (grey) and variant (black). **(C)** Correlation between high aneuploidy levels and high expression levels of histone genes calculated by Chi-squared test. The gene expression was defined as “high” or “low” based on the absolute expression value relative to the mean average. **(D)** Frequency of mutations in histone genes by location on amino acid residue of the protein. Underneath, a schematic representation of protein structure with domains of canonical and specific variants. Each dot represents one mutation and the colour indicates the degree of functional impact predicted by MutationAssessor. Pie charts show the proportion of mutation types for each gene across all cancers.

While histone genes are often overexpressed in cancer, mutations are less frequent (Shah et al., 2014; Ferrand et al., 2020; Ghiraldini et al., 2021). The most prominent example of cancer-associated histone mutations are *H3* missense mutations in gliomas (Schwartzentruber et al., 2012; Sturm et al., 2012; Wu et al., 2012; Pathania et al., 2017; Nacev et al., 2019), with analogous mutations in chondroblastomas (Fang et al., 2016), sarcomas (Lu et al., 2016), and head and neck tumours (Papillon-Cavanagh et al., 2017). However, thanks to the availability of large patient databases with high-throughput sequencing data, the identification of mutations in histone variants has grown considerably (Shah et al., 2014; Bennett et al., 2019; Nacev et al., 2019). Leveraging data from the TCGA cancer cohorts, the mutation frequency of canonical and variant histone genes is generally low, with few cancer-specific exceptions of high percentages in diffuse large B cell lymphomas (DLBL) and uterine carcinoma (UCEC) (Figure 4A). Regardless of canonical or variant, the majority of mutations are missense, followed by truncating mutations (Figure 4B). Among the most frequent mutated genes are canonical *H1* histones (Figure 4C). An association between mutation status (regardless of mutation type) and increased aneuploidy was found prevalently for canonical histones of the *H2A*, *H2B* and *H3* families, and only a few significant associations with variants (i.e., *H2AZ.1*, *H3.3B*, and *H3.5*) (Figure 4C). Certain histone genes were only associated with high levels of aneuploidy (i.e., where at least 50% of chromosome arms were altered), notably the *H3.3B* variant (Figure 4C). For a comprehensive list of aneuploidy-associated mutations, all mutations associated with high aneuploidy scores (>10) are available on the HistoPloidyDB website.

While the initial oncohistone alterations were first described within the tail region of histones, affecting their PTMs, mutations with functional consequences have also been reported within different histone domains (Bennett et al., 2019; Nacev et al., 2019). Figure 4D shows that the location of mutations in histone genes in the TCGA cancer cohort can occur throughout the gene body, however their predicted functional impact (as predicted by MutationAssessor) varies by domain. Gene-specific differences are notable, however the most impactful mutations map within tail or central fold domains (Figure 4D). In fact, the most prominent glioma-associated *H3.3* mutations produce a change from lysine to methionine (K27M and K36M) in either canonical *H3* or non-canonical *H3.3*, causing the inability to undergo crucial methylation modifications, thus perturbing the correct epigenetic marks genome-wide (Lewis et al., 2013). Interestingly, these effects are specific to methionine and not achievable by any other amino acid, as demonstrated by a reduction of H3K27 tri-methylation only with the methionine substitution in HEK293T cells (Castel et al., 2015), highlighting the importance of individual residues for the function of histones. In fact, the substitution to methionine produces structural changes that affect the ability to interact with other epigenetic factors, notably members of the epigenetic writer polycomb repressive complex 2 (PRC2), with major epigenetic reprogramming and repercussions on gene expression control (Lewis et al., 2013; Fang et al., 2016; Harutyunyan et al., 2019). A third class of notable *H3* mutations are G34R/V and G34R/W/L, which are associated with high-grade gliomas and bone tumours, respectively, and only occur in the *H3.3* variant gene (Behjati et al., 2013; Koelsche et al., 2017; Chen et al., 2020). Despite not being a PTM site *per se*, the G34 mutations affect the methylation status of the adjacent K36 residue, but only producing a local effect, as opposed to a genome-wide methylation effect by the K27M and K36M counterparts (Lewis et al., 2013; Zhang et al., 2017; Shi et al., 2018). A comprehensive analysis of the glioma mutations of canonical *H3* K27M, and *H3.3* variant mutations K27M and G34R revealed distinct interactomes and dysregulation of transcription and chromatin dynamics, further strengthening the array of potential oncogenic effects by disruption of single amino acid residues of histone genes (Siddaway et al., 2022). Interestingly, these mutations also exhibit precise clinical manifestations in anatomical location, age presentation, and cell of origin (Kallappagoudar et al., 2015; Bano et al., 2017). In the case of *H3.3* G34R/V mutations in gliomas, this specificity has been attributed to a developmental permissive chromatin context that allows the transformation of specific ontogeny, highlighting the potential variability in tumourigenic mechanisms by histone mutations, not only by the amino acids alone but also by cellular context (Chen et al., 2020).

The relationship between altered epigenetic landscapes and aneuploidy may be linked to the onset of abnormal transcriptional programmes affecting chromosome segregation, or aberrant chromatin specification of crucial genomic regions such as centromeres and telomeres, resulting in chromosomal instability (Herrera et al., 2008; Decombe et al., 2021). For example, H3G34R mutations display telomeric alterations potentially linked to altered methylation status of telomeric regions, where H3.3 is normally deposited (Lewis et al., 2010; Schwartzentruber et al., 2012; Sturm et al., 2012). The H3.3-H4 chaperone DAXX has also been implicated in epigenetic dysregulation and chromosomal stability in cancer (Tang et al., 2015; Mahmud and Liao, 2019). DAXX participates in the deposition of H3.3 at pericentromeric regions, and its loss has been linked to features of genomic instability (i.e., micronuclei, nuclear blebbing, and multinucleation) (Ishov et al., 2004; Morozov et al., 2017). *DAXX* mutations are prevalent in non-functional pancreatic neuroendocrine tumours (NF-PanNETs), where they correlate with disease severity and the presence of copy number variations (Hong et al., 2020) and are associated with altered global DNA methylation patterns (Pipinikas et al., 2015). Tumours with *DAXX* mutations also exhibit telomeric defects (Heaphy et al., 2011; Tang et al., 2015). Additional insights into epigenetics and aneuploidy come from observations of high aneuploidy rates in miscarriages during embryonic windows where critical epigenetic reprogramming (primarily DNA methylation) occurs (Nikitina et al., 2016; Tolmacheva et al., 2020), also supported by reports describing a deregulation of genes involved in cell cycle and histone modifications in placental tissue undergone pregnancy losses (Zhu et al., 2017). Moreover, DNA methylation patterns have been associated with aneuploidy phenotypes in cancer (Herrera et al., 2008; Ehrlich and Lacey, 2012). Similarly, it has been demonstrated that a disturbed acetylation status of histone H3 at K56 produced whole chromosome duplications and missegregation events in yeast, which was dependent on the activity of the histone chaperone ASF1 (Chan and Kolodner, 2012).

**FIGURE 5:**
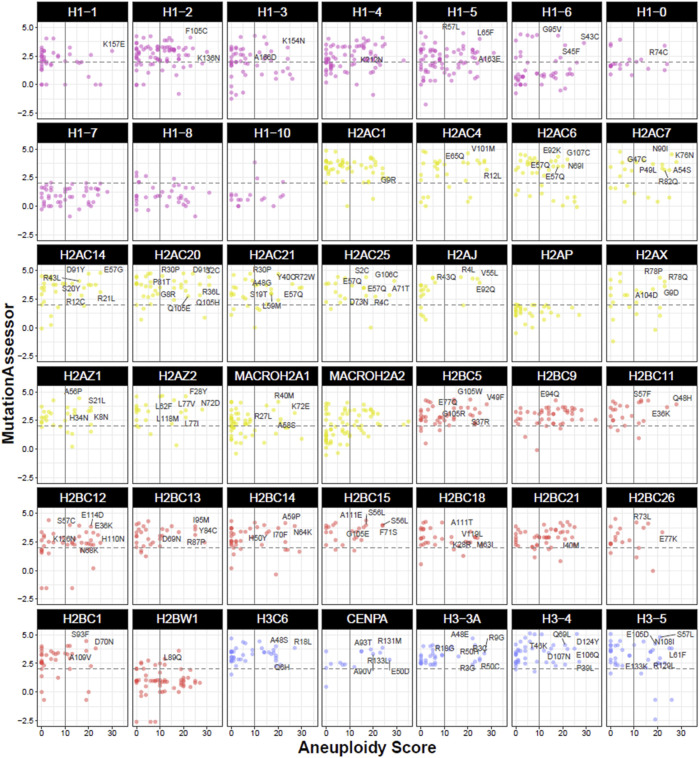
Functional and phenotypic properties of mutations in histone genes in the TCGA cohort. Dot plots represent individual mutations by gene and their associated Aneuploidy Score and predicted functional impact by MutationAssessor. Vertical intersect marks Aneuploidy Scores larger than 10 affected chromosome arms, while horizontal dashed intersect marks a medium functional impact score (>2) on MutationAssessor. Mutations falling within the top right quadrant indicate high aneuploidy and potential protein disruption, with some mutation details outlined in text. Colours indicate the histone family (H1, H2A, H2B, or H3). Data was obtained from cBioPortal in TCGA cancers from PanCancerAtlas (Cerami et al., 2012; Gao et al., 2013).

On the other hand, histone mutations within the histone fold domain, which are not primarily targeted by PTMs, may affect nucleosome stability and interactions with chromatin. In turn, these changes in chromatin accessibility can perturb gene expression regulation with oncogenic consequences (Arimura et al., 2018; Bennett et al., 2019), highlighting the importance of investigating histone mutations beyond PTM sites. Comparison of individual mutations against MutationAssessor scores and Aneuploidy Scores identified a wide range of gene alterations with an association with an aneuploid phenotype that also correlates with a functional defect, highlighting the H2A and H3 histone families with the most genes with both properties (Figure 5).

In terms of their impact on genomic stability, it was reported that diffuse gliomas without H3K27M mutations showed a lower degree of chromosomal aberrations compared to mutated patients (Dufour et al., 2020), and that both *H3.1* and *H3.3* mutations display highly unstable genomes by copy number alterations (Buczkowicz et al., 2014). The target of *H3.3* mutations flank a crucial Ser residue that is phosphorylated in early mitosis to ensure faithful chromosome segregation (Hinchcliffe et al., 2016). *In vitro*, mutants of *H3.3* (K27M) presented mitotic aberrations with increased rates of aneuploidy, both numerical (monosomies, tetrasomies, and WGD) and structural abnormalities (Mackay et al., 2017; Bočkaj et al., 2021). *H3.3A* mutations are associated with p53 alterations, chromosomal gains and losses (specifically loss of 17p), and a complex karyotype, when compared to *H3.1*-mutated cohorts (Castel et al., 2015). Clinical differences also exist between *H3.1* (canonical) and *H3.3A* (variant) mutations, with the latter being associated with disease aggressiveness and dismal prognosis (Castel et al., 2015). *H3.3A* and *H3.3B* mutations are also found in giant cell tumour of the bone where they are reported to behave as initiating events that can lead to the transformation of benign tumours into malignant forms. Malignant GCTBs are characterised by higher mutational rates and aneuploidy, which arise following *H3.3* mutations, suggesting a driver role in compromised genomic stability (Fittall et al., 2020). Indeed, in embryonic fibroblasts, mutations of *H3.3B* were found to be associated with increased aneuploidy (Bush et al., 2013).

Mutations in canonical genes of the linker histone *H1* have been reported in multiple myeloma, which has been associated with impacting on chromatin organisation and genomic stability (Hoang et al., 2018; Walker et al., 2018; Maura et al., 2019). Missense mutations in the conserved histone-fold domain in *H1*.*5*, *H1*.*3*, *H1*.*4*, and *H2BC12* were identified as drivers, due to the mutations occurring at key sites of nucleosome binding (Pawlyn et al., 2016; Maura et al., 2019). Interestingly, analogous mutations within the fold domain were also described in follicular lymphoma (Li et al., 2014; Krysiak et al., 2017) and chronic lymphocytic leukaemia (Landau et al., 2015). In particular, the lymphoma-associated Ser102Phe *H1.2* mutation showed a reduced ability to bind chromatin in mouse embryonic stem cells, indicating an improper function in the modulation of chromatin compaction (Okosun et al., 2014). In multiple myeloma, *H1.4* and *H1.2* mutations were identified as clonal, which suggests their involvement in initiating and/or maintaining the oncogenic status by affecting genomic stability, also supported by a high rate of structural and numerical aneuploidies and complex cytogenetic rearrangements (Pawlyn et al., 2016; Aksenova et al., 2021).

More mechanistic insights on the effects of *H1.4* mutations come from the homonymous *HIST1H1E* (*H1*.*4*) syndrome, characterised by frameshift mutations in the C-terminal tail of H1.4 causative of intellectual disability and multi-organ and skeletal abnormalities (Burkardt et al., 2019). Functionally, these mutations affect a critical portion of Ser/Thr residues that are phosphorylated to modulate chromatin compaction, particularly during mitosis (Hergeth and Schneider, 2015). *In vitro*, *H1*.*4* mutations led to more uncompact chromatin, replicative senescence, and decreased proliferation with increased susceptibility to DNA damage. Mutated fibroblasts also displayed nucleolar instability and a higher degree of aneuploidy compared to controls, indicating drastic changes in genomic stability through chromatin modelling by dysfunctional histone H1 (Flex et al., 2019). As described earlier, depletion of H1 in *Xenopus* is associated with aberrant chromosome segregation (Maresca et al., 2005).

While the *H2A* family is frequently upregulated and correlated with aneuploidy in terms of expression, a lower degree of mutations is observed in cancers (Figure 4A). This is particularly evident for variants of *H2A*, possibly due to their diverse and non-redundant roles, which could be incompatible with major functional defects. The only *H2A* member with a significant association with aneuploidy is *H2AZ.1* (Figure 4A), in agreement with the role of the H2A.Z variant in chromosomal stability (discussed above). In yeast, H2A.Z was shown to be required for sister chromatid cohesion for accurate chromosome segregation that is dependent on acetylation (Sharma et al., 2013), which could be compromised by mutations at PTMs sites. In particular, acetylation has been shown to be a crucial PTM for H2A.Z (Millar et al., 2006; Halley, 2009; Ishibashi et al., 2009). *H2AZ.2*-depleted (but not *H2AZ.1*) HeLa showed genomic instability in the form of micronuclei, chromatin bridges and lagging chromosomes; this role of H2AZ.2 in chromosomal stability was further shown to be dependent on PTMs, as mutated acetylation sites were unable to rescue the defects (Sales‐Gil et al., 2021). Moreover, some evidence of *H2A* mutations and chromosomal instability may come from reports on H2A.X, which is a crucial player in the DNA damage response cascade and a classical marker of double stranded DNA breaks (DSB) (Kuo and Yang, 2008). It has been proposed that H2A.X acts as a dosage-dependent “protector” of tumour-associated chromosomal aberrations (Bassing et al., 2003; Celeste et al., 2003; Franco et al., 2006). In fact, the 11q23 locus where *H2A.X* is located is deleted in a large number of malignancies (Srivastava et al., 2009) and genetic association analyses revealed that mutations in *H2A.X* predispose to a susceptibility to Non-Hodgkin lymphoma that is characterised by recurrent chromosomal translocations (Novik et al., 2007). Even a single allele deletion of *H2A.X* increases genomic instability and promotes tumourigenesis in p53 null mice. Homozygous deletions of *H2A.X* greatly increased the development of lymphomas and the occurrence of structural aberrations including chromosomal translocations and amplifications (Celeste et al., 2003). Structural aneuploidies (mostly translocations) in the context of *H2A.X* loss are the result of improper repair of DSBs in both intentional V(D)J-related breaks and generalised DNA repairs (Bassing et al., 2003).

Mutations of the *H2B* family have also been described in a wide range of malignancies [reviewed by Wan and Chan (Wan and Chan, 2021)], with delirious effects on nucleosome stability and chromatin accessibility. A notable example are E76K and E76Q mutations in the canonical *H2B* gene, which are located at regions of contact with other histones within the nucleosome, hence affecting the formation and stability of the nucleosomal octamer (Nacev et al., 2019). In turn, destabilised nucleosomes can lead to aberrant histone exchanges and altered chromatin accessibility with consequences on transcription of pro-oncogenic programmes (Arimura et al., 2018; Bennett et al., 2019; Bagert et al., 2021). In terms of *H2B* mutations affecting chromosome stability, *H2BC21* has been identified as a driver event in copy number alteration-based oncogenic events at pan-cancer level (Belikov et al., 2022). Among mutations that affect chromosomal stability, the G53D mutation in canonical *H2B* genes has been reported in different tumours (Bennett et al., 2019; Nacev et al., 2019), which occurs within the inner fold region of H2B, a site of ubiquitination that affects centromere stability and chromosome segregation in yeast (Maruyama et al., 2006). Similarly, it was shown that aberrant ubiquitination of H2B at Lys120 (via USP22 depletion) leads to mitotic defects, including micronuclei, nuclear abnormalities, and increase in chromosome gains and losses (Jeusset et al., 2021). Mutations at the acetylation site at Lys24 in *H2BC3* and *H2BC4* have been reported in the neoplastic Extramammary Paget’s disease (EMPD) of the skin (Takeichi et al., 2020). While patient numbers were too limited to draw statistically significant conclusions, both mutated cases showed particularly invasive disease (Takeichi et al., 2020), which is often associated with aneuploidy (Lloyd and Flanagan, 2000). *H2BC11* and *H2BC8* were also found to be mutated in ovarian carcinosarcoma patients with evidence of high rates of copy number variations (Zhao et al., 2016). Canonical *H2B* genes are also mutated at a high rate in follicular lymphoma, however the biological significance remains unclear (Krysiak et al., 2017).

## 4 Discussion

Histones and their variants coordinate crucial cellular processes, including chromosome segregation and the maintenance of genomic stability, which are of particular relevance in cancer. Cancer is characterised and fuelled by defective genomic health, which precedes and maintains states of large genomics rearrangements and aneuploidy. In recent years, histones have gained increasing interest in the development of malignancies. The regulation of quantity and functionality of histones in the cell is fulfilled at several levels, including transcriptional, post-transcriptional, translational, and post-translational levels. Therefore, the alterations of histones that can produce oncogenic effects can be multi-faceted. Here, we have reviewed the literature on the association between alterations in histones and aneuploidy, with the aid of publicly available TCGA cancer database. We gathered all correlations between expression, mutation, and copy number variations in histone genes with aneuploidy in the HistoPloidyDB database.

The majority of studies on histones and aneuploidy have focused on dysregulated expression and an imbalance between the incorporation of canonical and variants. Despite apparently contradictory reports on positive or negative correlation between expression levels and aneuploidy status (also evident from aneuploidy correlations in Figure 3B), it is likely that the overall effect is dependent on the net abundance of canonical and variant histones with respect to their baseline levels, as well as the availability and function of histone chaperones. Considering the differential effects on environmental cues and tissue-specificity, different tumours will display specific combinations of canonical/variant ratios, which can lead to genomic instability and aneuploidy. In summary, the dysregulated expression of histones may affect the balance between canonical and variant forms required to maintain chromosomal that may initiate or maintain tumourigenesis.

While less frequent than changes in gene expression, mutations in histones genes are also found in cancers and can be associated with aneuploidy. Interestingly, some histones only associated with high aneuploidy rates (Figure 4C), which may hint at different mechanisms producing chromosomal instability. This is of relevance functionally, as different scales of aneuploidy can have distinct effects on cellular functions (Weaver and Cleveland, 2009; Silk et al., 2013). In fact, aneuploidy is a double-edged sword in cancer, as it can be a deleterious or advantageous feature to cancer fitness and drug resistance; the exact role of aneuploidy in cancer is also still a topic of debate, with numerous contextual variables determining whether it promotes or suppresses tumourigenesis (Ben-David and Amon, 2020; Lukow and Sheltzer, 2022). Nevertheless, the heterogeneity of histone mutations, which are also not clustered within a particular protein domain (Figure 4D), does not allow for an accurate correlation with cellular phenotypes, but it can provide a platform to further characterise aneuploidy-associated mutations. As with the role of aneuploidy itself, the exact role of histones in tumourigenesis is not yet defined and may depend on the type and level of alteration to produce a specific outcome. Most histone mutations have been categorised as passenger (Bennett et al., 2019; Nacev et al., 2019), however further characterisation of functional effects of these alterations can uncover cooperating mechanisms.

Nevertheless, it appears that aberrations in histones is a yet undefined component of cancer, whether is a cause or a consequence. This is also supported by numerous reports of amplifications of the histone clusters loci in cancers, suggesting a functional advantage; for instance, the histone clusters loci are frequently subjected to copy number variation (mainly amplifications) in gastric cancer (Gorringe et al., 2005), in breast and basal cell carcinomas (Morelle et al., 2014), leukaemia (Holmfeldt et al., 2013; Simonetti et al., 2019), ovarian cancer (Zhao et al., 2016), and osteosarcoma (Sadikovic et al., 2009; Pires et al., 2022).

Overall, this work lays the ground for research into the role of histones and their variants in the disruption of chromosomal stability in cancer. For instance, comprehensive analyses of mutations in chromosome segregation genes have shed light on their contribution to genomic stability in health and disease states (Islam et al., 2022), highlighting the importance of investing functional effects of such alterations. Beside the fundamental and well-characterised contribution of the H3 variant CENP-A to the specification and maintenance of the centromere, the data collected altogether may indicate that other histone variants could also contribute to the specify and/or maintain a correct centromeric/kinetochore function. Future work will determine the exact role in oncogenesis, whether predisposing, promoting, causing, or maintaining the pathogenesis of the disease.

## Data Availability

The original contributions presented in the study are included in the article/Supplementary Material, further inquiries can be directed to the corresponding authors.
